# Optimizing Protein Profile, Flavor, Digestibility, and Microstructure: The Impact of Preheating and Reheating in Stir-Fried Chicken

**DOI:** 10.3390/foods14040643

**Published:** 2025-02-14

**Authors:** Kumayl Hassan Akhtar, Ziwu Gao, Zeyu Zhang, Xiangxiang Sun, Yumei Yu, Jiale Ma, Dequan Zhang, Zhenyu Wang

**Affiliations:** 1 Institute of Food Science and Technology, Chinese Academy of Agricultural Sciences, Beijing 100193, China; thekumayl@hotmail.com (K.H.A.); gzw96530@163.com (Z.G.); zhangzeyu@caas.cn (Z.Z.); 17302258026@163.com (J.M.); dequan_zhang0118@126.com (D.Z.); 2 Integrated Laboratory of Processing Technology for Chinese Meat and Dish Products, Ministry of Agriculture and Rural Affairs, Beijing 100193, China; 3 Shandong Provincial Key Laboratory of Animal Resistance Biology, Key Laboratory of Food Nutrition and Safety of Shandong Normal University, College of Life Sciences, Shandong Normal University, Jinan 250014, China; sxxiang@sdnu.edu.cn; 4 School of Food and Health, Beijing Technology and Business University, Beijing 100048, China; 17861903886@163.com

**Keywords:** reheating, doneness levels, GC-MS, protein digestibility, microstructure

## Abstract

Preheating and reheating play key roles in enhancing the nutritional and sensory qualities of stir-fried chicken. Thus, this study investigated the effect of preheating (D1) and reheating (D30) after 30 days of storage on the protein profile, lipid oxidation, flavor, texture, color, sensory properties, protein digestibility, and microstructure of household stir-fried chicken. Four doneness levels of preheating (medium rare (T1 = 62 °C), medium (T2 = 65 °C), medium well (T3 = 68 °C), and well done (T4 = 71 °C) and reheating (72 °C) were selected. Results revealed that preheating and reheating enhanced protein and lipid oxidation, while the unfolding of α-helix improved quality and digestibility. GC-MS identified 59 volatile compounds, including β-phellandrene and 1-octen-3-ol in preheating and anethole and benzaldehyde in reheating. Texture analysis showed decreased hardness, chewiness, and gumminess at T3. Lightness (L*) and redness (a*) were largely unaffected, except for the well done (D1T4, D30T4) doneness level, although yellowness (b*) increased with the increasing doneness levels. Sensory evaluation highlighted improved appearance, tenderness, and overall acceptability, particularly for D30T3. Digestibility enhanced with reheating, reaching up to 80% intestinal digestibility at D30T3. Microstructural analysis unveiled structural deformation, which was most pronounced at T4. Preheating followed by reheating enhanced meat quality attributes, with D30T3 emerging as the optimal doneness level for the industrial production of stir-fried chicken.

## 1. Introduction

The modernization of the food industry and catering sector has popularized the use of prepared dishes, including traditional Chinese cuisine like stir-fried chicken, for convenience, affordability, and efficiency [1]. Stir-fried chicken is considered healthy due to its low fat content, low cholesterol content, and high protein content [2]. Frying transforms the chicken by giving it a crispy exterior and tender interior, making it a preferred cooking method among people of all ages [3]. Usually, stir-fried chicken is commercially prepared, refrigerated, and sold as ready-to-eat food.

Stir-fried chicken benefits significantly from proper preheating and reheating techniques. Preheating ensures the even cooking of meat, while reheating involves heating precooked food that is stored for a few days to several weeks [4]. Recently, the reheating of pre-cooked meat has received special attention from consumers because of its convenience, although it has been used as a preservation method for many years [5]. Reheating methods like microwaving, electric grilling, steaming, and boiling have been extensively applied by small to medium-sized businesses to their products [6]. Additionally, reheating processes are used by Chinese astronauts during their space journeys [7]. Several studies have analyzed the quality and sensory characteristics of meat and meat products during reheating and found that better results were achieved by applying reheating [2,4,8]. Protein degradation, lipid oxidation, and the Maillard reaction contribute to improving tenderness, juiciness, and other textural attributes of reheated meat [9]. Previous findings have shown that frying retains fat-soluble nutrients while boiling preserves water-soluble nutrients [10,11]. Therefore, the diced breast cubes can be stir-fried at a certain doneness level and reheated by boiling to maintain the nutritional profile of stir-fried chicken to obtain the desired nutrient-rich, flavored product.

Flavor is one of the essential characteristics of stir-fried chicken. Volatile organic compound (VOC) analysis in meat dishes is critical to identifying the flavor quality. Compared to other techniques, GC-MS is a sensitive, specific, and reliable method containing extensive mass spectra libraries of volatile compounds [12]. Another important parameter is digestibility, which involves a complex enzymatic process that digests meat [13]. Recently, in vitro protein digestibility was studied to understand how much protein is digested at the gastric and intestinal phases [14]. Studies reported on protein digestibility in meat; however, their focus was either on preheating or on reheating, instead of preheating and reheating together and at different doneness levels [15,16,17].

In the food industry, meat dishes are often fully cooked, frozen, and reheated by end consumers, which can compromise their quality and digestibility due to excessive protein denaturation, moisture loss, and lipid oxidation [18]. Recently, there has been a shift toward medium-cooked preheated meat dishes to meet the growing demand for convenient, ready-to-cook food [19]. Additionally, central kitchens have gained positive traction among restaurants for such foods, enabling consistency and efficiency in meal preparation [20]. However, the interplay between preheating and reheating at specific doneness levels remains underexplored, particularly for dishes like stir-fried chicken.

Previous researchers studied different cooking methods [21,22,23], some assessed doneness levels [24,25], while some conducted significant research on preheating and reheating [5,8,26] of chicken. However, the effect of preheating and reheating, together at particular doneness levels (medium rare, medium, medium well, and well done), on the protein profile, flavor, digestibility, and microstructure of stir-fried chicken has not been discussed. Therefore, this study was designed to investigate the effect of preheating and reheating, focusing on the eating quality, flavor, and digestibility of stir-fried chicken. Additionally, protein and lipid oxidation, sensory evaluation, and microstructures were evaluated and correlated to figure out the appropriate doneness level.

## 2. Materials and Methods

### 2.1. Materials and Chemicals

Whole chicken carcasses (*n* = 18) weighing 1200 g ± 30 were purchased from the company (Hebei Meikeduo Foods Group, Tangshan, China), transported under controlled temperatures, and stored at −18 °C until the experiments. All chickens shared identical biological backgrounds, were fed the same diet, and were of the same age. Chickens were slaughtered following standardized procedures.

Standard 2-methyl-3-heptanone was acquired from LGC Biosearch Technologies Co., Ltd., Beijing, China. Pepsin and trypsin (from porcine pancreas) were purchased from Sigma-Aldrich, Madison, WI, USA. All other reagents and chemicals were acquired from Solarbio Life Sciences (Beijing, China) and Shanghai Aladdin Biochemical Technology Co., Ltd. (Shanghai, China).

### 2.2. Sample Preparation

**Figure 1:**
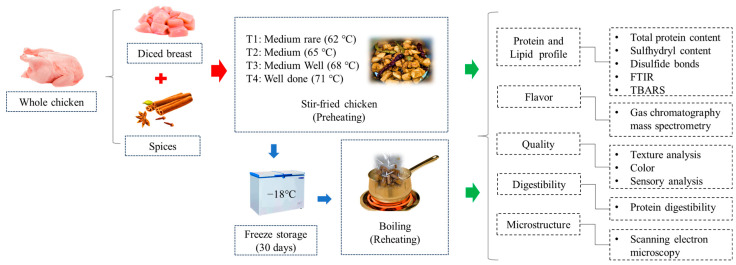
Simplified schematic diagram of experimental design.

Chicken breasts (250 ± 10 g) were removed from the carcass in the laboratory, diced into small pieces (2 cm × 2 cm × 2 cm), and thoroughly mixed. For each experiment, diced breast cubes were randomly divided into four equal portions (240 ± 0.2 g), with each portion corresponding to one cooking temperature. Cooking was performed in triplicate at four doneness levels for a total of 240 × 4 × 3 g. For 1000 g of meat, the ingredients added were fragrant leaves (4.5 g), aniseed (4.5 g), red chili (10 g), salt (8.3 g), ginger (20 g), soy sauce (20 mL), and oil (120 mL). Diced breast cubes were cooked in a wok placed on an induction plate (1400 W), according to the relevant degree of doneness of stir-fried chicken, following the methodology of Torun et al. [27] and Cross et al. [28]. Medium rare (62 °C), medium (65 °C), medium well (68 °C), and well done (71 °C) doneness levels were denoted as T1, T2, T3, and T4, respectively. The time required to achieve the doneness levels was 240 s, 280 s, 320 s, and 360 s, respectively. The listed temperatures represent the internal temperature of the meat at which cooking was halted. After preheating, samples were packed in a vacuum bag, labeled, and sealed. Half of the preheated samples were analyzed on the same day, while others were stored for 30 days at −18 °C and kept aside for reheating. After thawing at room temperature, reheating was performed by boiling the meat packed in vacuum bags until it reached a core temperature of 72 °C and subsequently cooling it for further analysis. During preheating and reheating, the temperature was frequently checked using a multi-channel temperature monitor device (LK-U type, Changzhou Blue Light Electronics Co., Ltd., Changzhou, China). The preheated and reheated samples were denoted as follows: D1T1 = preheated medium rare; D1T2 = preheated medium; D1T3 = preheated medium well; D1T4 = preheated well done; D30T1 = reheated medium rare; D30T2 = reheated medium; D30T3 = reheated medium well; D30T4 = reheated well done. The simplified schematic diagram of the experimental design is shown in Figure 1.

### 2.3. Total Protein Content

Stir-fried chicken sample (1 g) was homogenized with 10 mL of sodium decodyl sulfate (2% *w*/*v*), followed by centrifugation at 4000× *g* for 20 min. The supernatant was collected, and protein concentration was determined by BCA Protein Assay Kit (Thermo Fisher Scientific, Waltham, MA, USA).

### 2.4. Total Sulfhydryl (SH) Content

SH content was determined using methods described by Gao et al. [29]. The protein solution (2 mg/mL, 0.5 mL) was mixed with a Tris-Gly-8M urea buffer (2.5 mL) and Ellman’s reagent (DNTB) (4 mg/mL, 0.02 mL) and incubated at 25 °C for 30 min. The absorbance was recorded at 412 nm, while SH content was calculated using the following formula (Equation (1)).

(1) $$Total\;SH\;content\;\left(\frac{\mu mol}{mg}protein\right)=73.53\times A_{412}\times \frac{D_{1}}{C}$$

where *D*_1_ is the dilution coefficient (6.04) and *C* (mg/mL) is the protein content.

### 2.5. Disulfide Bonds Detection

Disulfide bonds were detected using the method described by Gao et al. [29]. The protein solution (2 mg/mL, 0.2 mL) was mixed with Tris-Gly-10M urea buffer (2.5 mL) and β-mercaptoethanol (0.02 mL). This was followed by incubation (25 °C, 60 min). After centrifugation for 10 min (30,000× *g*), the pellet was suspended in a mixture containing Tris-Gly-8M urea buffer (3 mL) and DNTB (0.03 mL). The absorbance was recorded (412 nm), and disulfide bonds were calculated using the following formula (Equation (2)).

(2) $$Disulfide\;bonds\;\left(\frac{\mu mol}{mg}protein\right)=73.53\times A_{412}\times \frac{D_{2}}{C}-SH\;content\;$$

where *D_2_* is the dilution coefficient (15) and *C* (mg/mL) is the protein content.

### 2.6. FTIR

The change in the protein secondary structure content was analyzed using a modified version of the method described by Gao et al. [29]. Cooked samples (5.0 g) were freeze-dried and subsequently ground into a fine powder. The ground samples were then mixed with KBr and scanned using a diamond attenuated total reflectance Fourier transform infrared spectroscopy machine (ATR-FTIR, BRUKER TENSOR 27). The scanning parameters were as follows: scanning range—400–4000 cm^−1^; number of scans—64; scanning rate—0.63 cm/s; resolution—32 cm^−1^.

### 2.7. Lipid Oxidation (TBARS)

Oxidation in lipids was determined using 2-thiobarbituric acid reactive substances (TBARS) following the method described by He et al. [30]. First, samples (2 g) were mixed with trichloroacetic acid (17.5%, *w*/*v*, 10 mL) and homogenate and then filtered. Then, 0.02 M 2-thiobarbituric acid (5 mL) was mixed with 5 mL filtrate and incubated in the water bath (100 °C, 40 min). After cooling, the solution was centrifuged at 4 °C (2000× *g*, 5 min). The supernatant (5 mL) obtained was mixed with dichloromethane (5 mL). The upper layer was used for absorbance at 532 nm and 600 nm, and the following formula (Equation (3)) was applied for calculation.

(3) $$TBARS\;\left(\frac{mg}{kg}\right)=\frac{\left(A_{532}-A_{600}\right)}{155}\times \left(\frac{1}{2}\right)\times 72.06\times 1000\;$$

### 2.8. Extraction and Quantification of Volatiles

Aroma compounds were analyzed and quantified by solid-phase micro-extraction gas chromatography–mass spectrometry (SPME-GC-MS) according to the methods of Sun et al. [31] and Marsol-Vall et al. [32]. First, the sample was minced, weighed (3 g), and placed into a transparent 20 mL headspace vial containing a PTF silicon stopper. Subsequently, an internal standard (1.5 μL) was prepared by adding 2-methyl-3-heptanone (1.68 μg/μL) in methanol, and the vial was sealed. Immediately, the vial was placed into the fully automated designated GC-MS space (PAL-RTC, Zwingen, Switzerland).

SPME-GC-MS (8890 GC, 5977B GC/MSD, Agilent Technologies, Santa Clara, CA, USA) was run to extract, separate, and identify the volatile compounds in stir-fried chicken. The conditions of GC-MS were as follows: (i) we used a DB-Wax capillary column (30 m × 320 μm × 0.25 μm); (ii) helium (99.99% pure) was used as a carrier gas with a flow rate of 1.0 mL/min; (iii) the front inlet temperature was 25 °C; (iv) the program temperature was 40 °C for 3 min, 70 °C at a rate of 2 °C/min, 130 °C at a rate of 3 °C/min, 230 °C at a rate of 10 °C/min, and 230 °C for 10 min; (v) there was a split ratio of 1:1 (*v*/*v*) for MS and OPD C200; (vi) the MS temperature was 230 °C. The NIST (20.0) library database was used to compare and identify volatile compounds. *LRI* was analyzed by performing internal standard RI detection under the same conditions with the following equation (Equation (4)):

(4) $$LRI=100n+\frac{100\left(t_{x}-{\;t}_{n}\right)}{t_{n+1}-t_{n}}$$

where *t_x_, t_n_*, and *t_n +_*_1_ are the retention times of compound *x*, alkane *n*, and alkane *n +* 1, respectively.

For VOC content determination, the following equation (Equation (5)) is used:

(5) $$C_{v}=\frac{S_{v}}{S_{a}}\times C_{a}$$

where *S_a_* and *S_v_* are the peak areas of standard (2-methyl-3-heptanone) and VOCs, while *C_a_* is the standard’s content.

### 2.9. Texture Profile Analysis (TPA)

TPA was measured using a Stable Micro Systems texture analyzer (TA-XT plus, 42432, London, UK) according to the work of Pan et al. [33]. Samples were sliced into (1 cm × 1 cm × 1 cm) cubes. A P50 probe was used to determine the texture, with two times compression at an interval of 5 s. The force applied was 20.0 g, while the speeds were 5.0 mm/s, 2.0 mm/s, and 2.0 mm/s as pretest, test, and posttest speeds, respectively.

### 2.10. Instrumental Color

The instrumental surface color (L*, a*, b*) was measured by a colorimeter (Konica Minolta, CM-600d1, Tokyo, Japan). We used specifications that included the following: pulsed xenon lamp, diffused illumination/8° view angle, measurement area; Ø8 mm, wavelength; 400–700 nm. First, colorimeter calibration was performed manually using a white calibration plate. Surface color was measured three times at three points of meat selected randomly, and results were taken as the average of the measured values [34].

### 2.11. Sensory Evaluation

Sensory evaluation was conducted by trained professionals and meat science research team members (*n* = 10), with an equal male/female ratio. Before evaluation, consent was obtained from the panelists, and then random samples were distributed to the tasters to understand the evaluation process. Each taster was provided with a sample labeled with a three-digit random number. Tasters evaluated the meat through sensory evaluation Performa designed according to the work of Hou et al. [35]. The Performa was divided into five columns ranging from 0 to 10, where 0 < x ≤ 2 was very poor while 8 < x ≤ 10 was an excellent sensory score. The sensory evaluation Performa is described in Table S1. Water and crackers were distributed to rinse the mouth and change the taste perception.

### 2.12. Protein Digestibility

The protein digestibility of preheated and reheated meat was calculated using the static in vitro digestion method with some modifications [2,36]. Briefly, 2.0 g of grounded meat sample was homogenized in 8 mL of double-distilled water on ice. The speed of the homogenizer was 10,000 rpm for 6 × 300 s, with a cooling interval of 30 s. For gastric digestibility, pepsin (0.064 g) was added at the ratio of 1:31.25 (*w*/*w*) after adjusting pH to 2.0 with 1 M of HCL solution. After that, the mixed sample was incubated at 37 °C for 120 min, where continuous shaking was performed to mimic the digestion process. For intestinal digestibility, trypsin (0.04 g) was added at 1:50 (*w*/*w*) after adjusting the pH to 7.0 with 1 M NaOH solution. The sample was incubated with continuous shaking. After 120 min, pepsin and trypsin added samples were inactivated by heating at 95 °C for 5 min. After inactivation, mixtures were deproteinized by adding ethanol (3:1) and stored at 4 °C for 12 h. The digest obtained was centrifuged at 10,000× *g* for 20 min at 4 °C. Precipitates were dried using a freeze dryer (LGJ-10 four-ring freeze-drying machine, Beijing, China) and weighed. Finally, results were presented as the degree of digestibility using the following Equation (6):

(6) $$DT=\frac{W_{t}-W_{i}}{W_{t}}\times 100$$

where *DT* is the digestibility, *W_i_* is the weight of dried insoluble protein, and *W_t_* is the total weight of the meat before digestion.

### 2.13. Microstructure

The microstructure of stir-fried chicken was analyzed following the method of Liu et al. [37], with minor modifications. A sample piece (1 cm × 1 cm × 0.5 cm) was cut from the cooked diced chicken and fixed in 2.5% glutaraldehyde, prepared in 0.1 mol/L phosphate buffer (pH 7.3), for 4 h. Subsequently, the samples were dehydrated sequentially in ethanol solutions of increasing concentrations (10%, 30%, 50%, 70%, 80%, 90%, 95%, and 100%) for 15 min. The dehydrated samples were freeze-dried immediately after being flash-frozen in liquid nitrogen. The samples were then fragmented into transverse sections and mounted on aluminum stubs. This was followed by gold coating. SEM observations were performed at an increased voltage of 10 kV, with images captured at magnifications of ×200 and ×500 using an SU-8010-HITACHI scanning electron microscope.

### 2.14. Statistical Analysis

Statistical analysis was performed using ANOVA with SPSS 27.0 software (SPSS Inc., Chicago, IL, USA). Experiments were conducted in triplicate, and the data were expressed as mean ± standard deviation. Duncan’s test was used to evaluate the significant differences at *p* < 0.05. MetaboAnalyst (https://genap.metaboanalyst.ca/MetaboAnalyst/, accessed on 12 November 2024) was used for VOC analysis. The correlation heat map was plotted using Pearson’s correlation analysis, with significance set at *p* < 0.05. Graphs were generated through the OriginPro 2024 learning edition (OriginLab Corporation, Northampton, MA, USA) and Microsoft Excel 2021.

## 3. Results and Discussion

### 3.1. Analysis of Protein Profile and Oxidative Stability

#### 3.1.1. Analysis of Total Protein Content

**Figure 2:**
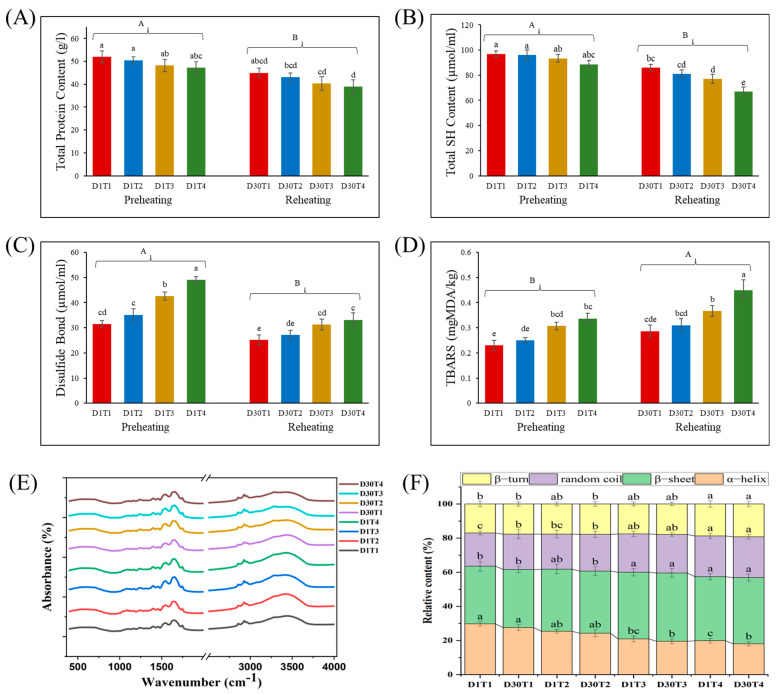
Total protein content (**A**). Total SH content (**B**). Disulfide bond (**C**). TBARS (**D**). FTIR absorbance (%) (**E**). Relative content (%) of protein (**F**) after preheating (D1) and reheating (D30) at four doneness levels. Lowercase a, b, c, d, and e show a significant difference (*p* < 0.05) among four doneness levels while A and B show a significant difference (*p* < 0.05) between preheating and reheating. D1T1 = preheated medium rare; D1T2 = preheated medium; D1T3 = preheated medium well; D1T4 = preheated well done; D30T1 = reheated medium rare; D30T2 = reheated medium; D30T3 = reheated medium well; D30T4 = reheated well done.

Figure 2A represents the total protein content in the preheating (D1) and reheating (D30) of stir-fried chicken. No significant differences were perceived among doneness levels. However, a decrease in protein content was noted as the cooking temperature increased. This reduction was attributed to the higher temperatures, which promoted protein denaturation, thereby reducing the availability of total protein content [38]. Reheating further reduced the protein content due to excessive protein denaturation, and a significant difference was observed between the two heating methods (49.43 g/L to 41.81 g/L). Furthermore, the Maillard reaction, initiated during heating and exacerbated during reheating, led to the formation of non-protein nitrogenous compounds, which reduced the total available protein content in the samples. Similar observations were reported by Wang et al. [1] when they reheated prepared chicken breast preheated at different cooked values. However, Nanje et al. [11] initially observed a high protein content after reheating using various cooking methods, which was possibly due to excessive moisture loss and the short storage time between preheating and reheating. Their findings showed a decrease in protein content after 3 days of storage.

#### 3.1.2. Changes in Total Sulfhydryl (Thiol) Content

Sulfhydryl (SH) content is the essential indicator of protein oxidation, influencing the texture and nutrition of the meat. Figure 2B represents the SH content when preheating (D1) and reheating (D30) at four doneness levels. As the doneness level increased from medium rare to well done, a decrease in the SH content was observed, indicating the promotion of protein oxidation. No significant difference (*p* > 0.05) was observed for preheating, but for reheating, the doneness levels were significantly different (*p* < 0.05), showing a reduction from 85.87 µmol/mL to 66.87 µmol/mL from medium rare reheated (D30T1) to well done reheated (D30T4) samples. Additionally, reheating resulted in a more substantial decline in total SH content than preheating (77.65 µmol/mL, 93.74 µmol/mL). This reduction can be attributed to the reactive oxygen species (ROS) generated during heating, which target sulfhydryl groups and lead to significant losses through oxidation reactions [29]. These findings align with the work of Parvin et al. [23], who found that boiling precooked beef meatballs reduced the thiol content. Similar findings were observed by Kim et al. [39] via the microwave reheating of ready-to-eat pork patties, which were roasted at 71 °C. Both studies showed that reheating decreased the thiol content regardless of the pretreatment or heating methods used. Furthermore, heating-induced denaturation is responsible for the cross-linking formation of disulfide bonds, ultimately decreasing the total SH content [1].

#### 3.1.3. Disulfide Bond (Protein Cross-Linking) Detection

Disulfide bond detection, which depicts the cross-linking in meat protein, is illustrated in Figure 2C. The results illustrated that disulfide bond detection significantly increased (49.01 µmol/mL) at higher doneness levels during preheating (D1) and reheating (D30). This phenomenon can be attributed to the increased exposure of proteins to heat and oxygen, which accelerates protein oxidation and the formation of disulfide bonds [40]. Interestingly, reheated samples exhibited considerably reduced disulfide bond content (33.16 µmol/mL) compared to the preheated samples, despite the crosslinking that occurred. Similar findings were reported by Ferreira et al. [16], who observed a decrease in the disulfide bond in chicken patties reheated after 7 days of storage. It is worth noting that thiol oxidation occurred during reheating, but that the mechanism for disulfide bond formation may be different from that seen in the typical thiol-to-disulfide bond conversion. This needs further investigation [16]. Meanwhile, reheating broke down the disulfide bond and released free thiols that were oxidized by free radicles [41]. Furthermore, disulfide bonds contribute to the formation of insoluble protein aggregates, leading to toughening and shrinkage of meat, as observed by TPA [42].

#### 3.1.4. Protein Secondary Structure Analysis

FTIR was employed to measure the protein secondary structure, as depicted in Figure 2E. The alteration in the shape of the Amide I (1600–1700 cm^−1^) and Amide II (1500–1600 cm^−1^) bands was attributed to the stretching vibrations of C=O and C-N bonds [29]. These changes were noticeably observed at the preheating and reheating of stir-fried chicken. The peak intensities were higher during preheating and decreased upon reheating, reflecting the denaturation of the protein secondary structure. Gong et al. [15] observed lower wave numbers with the increase in the core temperature of beef. At doneness levels D1T3 and D30T3, moderate peaks were observed, indicating that medium well cooking (T3) preserved the integrity of the protein secondary structure. Similarly, weak absorption bands were observed between 2850 and 2950 cm^−1^, corresponding to methyl and methylene groups, and the asymmetrical stretching of the carbonyl group in triglyceride ester bonds decreased with increased heating [43]. However, excessive heating (T4) can transform the protein structure into a more stable state where no further denaturation occurs [44].

**Figure 6:**
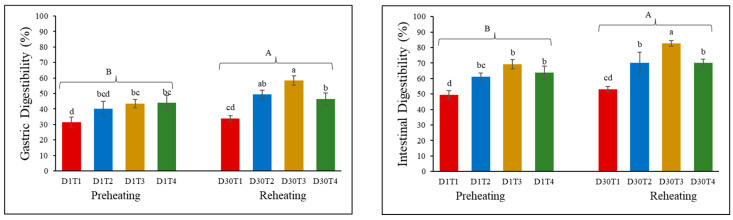
Gastric and intestinal digestibility (%) of preheating (D1) and reheating (D30) at four doneness levels. Lowercase a, b, c, and d show a significant difference (*p* < 0.05) among four doneness levels, while A and B showed a significant difference (*p* < 0.05) between preheating (D1) and reheating (D30). D1T1 = preheated medium rare; D1T2 = preheated medium; D1T3 = preheated medium well; D1T4 = preheated well done; D30T1 = reheated medium rare; D30T2 = reheated medium; D30T3 = reheated medium well; D30T4 = reheated well done.

**Figure 7:**
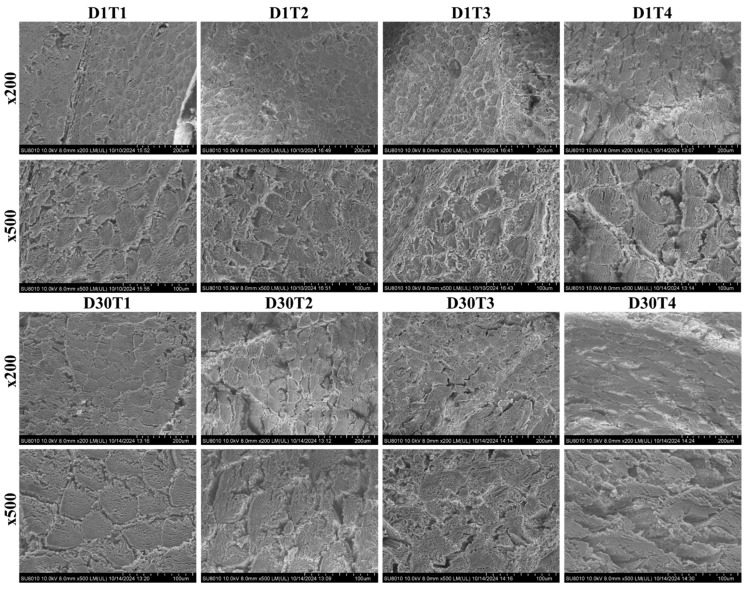
Microstructure analysis of preheating (D1) and reheating (D30) at four doneness levels at ×200 and ×500 magnification. D1T1 = preheated medium rare; D1T2 = preheated medium; D1T3 = preheated medium well; D1T4 = preheated well done; D30T1 = reheated medium rare; D30T2 = reheated medium; D30T3 = reheated medium well; D30T4 = reheated well done.

The quantitative analysis of the protein secondary structure revealed significant (*p* < 0.05) changes during preheating and reheating (Figure 2F). Preheating reduced α-helix content while increasing the presence of random coil, β-sheet, and β-turn structures, reflecting protein unfolding and aggregation, particularly at D1T3 and D1T4. Reheating, however, stabilized secondary structures, with minimal changes observed. The reduction in α-helix content due to unfolding was accompanied by an increase in random coil, attributed to weakened hydrogen bond stretching vibrations. This unfolding led to structural deformation that kept the water inside, improving the texture of stir-fried chicken. The effect was more pronounced during frying, as illustrated by Abd Rashid et al. [43]. Preheating at a lower temperature and then reheating allows actin and myosin peptide chains to react with digestive enzymes to improve meat’s digestibility. At higher temperatures (T4), myosin peptide chain polymerization via disulfide bonds led to macromolecule formation [45], reducing solubility to digestive enzymes, as shown in the protein digestibility results (Figure 6). The observed shift from ordered to disordered secondary structures, with the highest level of disorder seen at T4, correlated with microstructural changes, showing significant myofibrillar deformation at T4 (as observed in Figure 7). The increase in β-sheet content, consistent with meat hardness [44], aligned with the TPA results shown in Table 1.

#### 3.1.5. Lipid Oxidation (TBARS) in Stir-Fried Chicken

The TBARS content, which indicates lipid oxidation, alters the flavor, nutrition, and shelf life of stir-fried chicken, forming secondary oxidation products, primarily malondialdehyde (MDA), which serves as a marker for lipid oxidation. Figure 2D shows the MDA contents in the preheating (D1) and reheating (D30) methods. TBARS values of stir-fried chicken presented a trend of a significant increase during preheating and reheating. The well done doneness level showed higher MDA content (0.33 mgMDA/kg) than other samples. This was primarily due to the increased heating temperature. Subsequently, the level of MDA was higher in reheated (0.35 mgMDA/kg) than in preheated (0.28 mgMDA/kg) samples, which was consistent with the findings of Li et al. [38]. Similar findings were observed by Zhao et al. [46] using duck breast meat reheated by boiling after 7 days of storage. This phenomenon can be attributed to repeated heating, which accelerates lipid degradation and partial oxidation during preheating [47]. A high MDA content could increase oxidative stress and cause serious health concerns because of its carcinogenic effect [48]. Furthermore, the free radicles generated during preheating result in a chain reaction of lipid oxidation, producing further TBARSs [49]. Notably, ROS and natural antioxidants (such as vitamin E) are inactivated and depleted during the heating process, exaggerating lipid oxidation. The decrease in natural antioxidants reduces meat’s ability to neutralize oxidative stress, leading to the loss of essential fatty acids (such as omega-3) [50]. However, 1–2 mgMDA/kg is considered the threshold level of TBARS [51], and the TBARS values of the present study were below this limit.

### 3.2. Analysis of VOCs by SPME-GCMS

Cooked meat is defined by the flavor produced by VOCs. A total of 59 volatile compounds were detected in the preheating and reheating of stir-fried chicken, including hydrocarbons (17), aldehydes (11), ketones (7), esters (6), furans (1), alcohols (14), ethers (2), and another (1) compound. During preheating, 47 VOCs were identified, while during reheating, the number of VOCs fell to 43. The radar map showed these volatile compounds according to their chemical characterization as regard preheating and reheating methods (Figure S1A). Hydrocarbons and alcohols were the most abundant volatile compounds during preheating and reheating, following aldehydes and ketones. Most of the aldehydes, ketones, and alcohols were found for the D1T2 sample, the largest number of esters accounted for the D1T3 sample, and the majority of hydrocarbons were observed for the D1T4 sample. On the other hand, in Figure S1A, the D30T2 and D30T4 samples had the most hydrocarbons, the D30T3 sample had the most ketones and esters, and alcohols were higher in the D30T2 sample after the reheating method. This could be because, during storage, VOCs undergo oxidation and degradation reactions, while reheating redistributes and sublimates VOCs [52].

**Figure 3:**
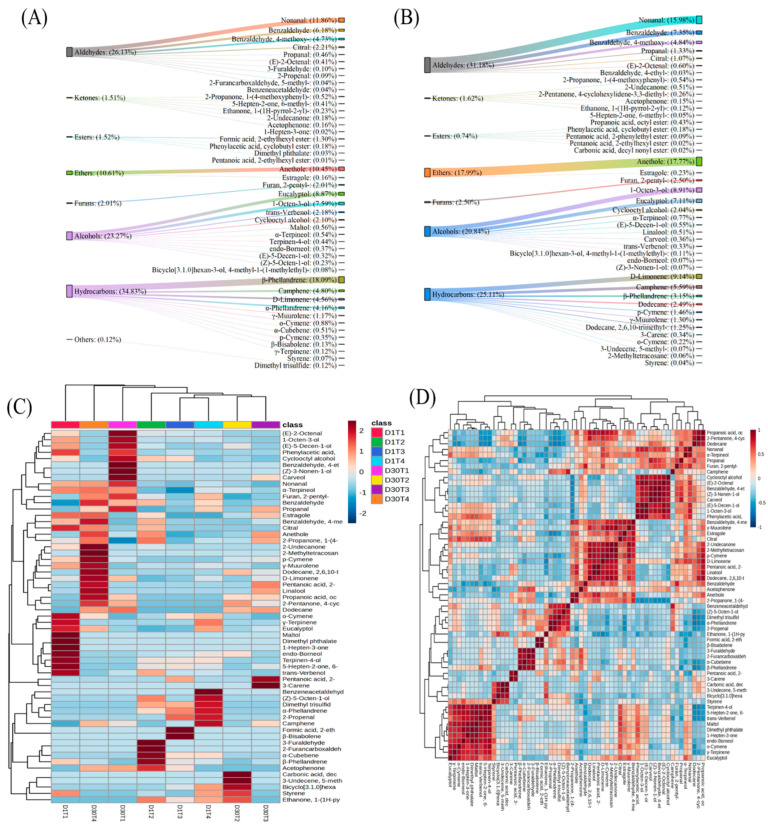
The concentration of volatile compounds at preheating (D1) (**A**) and reheating (D30) (**B**) and a heat map (**C**) showing the volatile compounds at preheating (D1) and reheating (D30) at four doneness levels. A correlation heat map (**D**) of all volatile compounds identified. D1T1 = preheated medium rare; D1T2 = preheated medium; D1T3 = preheated medium well; D1T4 = preheated well done; D30T1 = reheated medium rare; D30T2 = reheated medium; D30T3 = reheated medium well; D30T4 = reheated well done.

The concentration of VOCs indicates their importance in flavor production. The change in their concentration during preheating (D1) and reheating (D30) is illustrated in Figure 3A,B. Hydrocarbons were the most abundant VOCs, with a concentration of 30.04%, suggesting they had a significant role in flavor production in stir-fried chicken. On average, the other VOC concentrations were as follows: aldehydes, 28.61%; alcohols, 22.07%; ethers, 14.24%; and others, 5.01%. The content of all volatile compounds increased after reheating, except for alcohols and hydrocarbons. Wang et al. [53] showed that the decrease in alcohols and hydrocarbons was due to their oxidation during stir-frying. In the present study, the concentration of hydrocarbons dramatically decreased from 34.83% to 25.11%, although the number of hydrocarbons (12) remained the same. The reason behind this could be due to their high volatility and their chemical reactions with VOCs, which led to the production of other compounds [54]. A decrease in the number of aldehydes was observed during reheating, but overall, the content rose from 26.13% to 31.18%, improving the aroma characteristics of stir-fried chicken.

The Venn diagram shows similar volatile compounds at preheating (D1) and reheating (D30) (Figure S1B). During preheating, the number of common volatile compounds was 18. In contrast, during reheating, the number volatile of compounds was reduced to 14. The decrease in common volatile compounds was due to the production of new VOCs during reheating, highlighting the breakdown of VOCs produced during preheating [55].

The heat map was generated to illustrate the concentration of each volatile compound at different doneness levels and after using different heating methods (Figure 3C). The map showed that characteristic volatile compounds were detected during reheating, especially hydrocarbons, and esters, which were mainly produced by the Maillard reaction and thermal degradation [54]. In reheating, D30T1 and D30T4 exhibited new VOCs that significantly influenced flavor production. Among VOCs, the highest contents were observed for nonanal, anethole, eucalyptol, and β-phellandrene during preheating. In reheating, along with nonanal and anethole, 1-octen-3-ol and d-limonene contributed significantly to the VOCs in stir-fried chicken. Nonanal, anethole, and eucalyptol contributed fruity, sweet, and minty flavors, improving the aroma during preheating, while 1-octen-3-ol and d-limonene contributed to fresh umami flavors, boosting the overall sensory profile of the meat in reheating. It is worth noting that α-cubebene, α-phellandrene, γ-terpinene, (Z)-5-octen-1-ol, benzene acetaldehyde, dimethyl phthalate, dimethyl trisulfide, and terpinen-4-ol were only identified during preheating and 2-methyltetracosane, 2-pentanone, 3,3-diethyl-4-cyclohexylidene, 5-methyl-3-undecene, 3-carene, (Z)-3-nonen-1-ol, 4-ethyl-benzaldehyde, carbonic acid, decyl nonyl ester, carveol, dodecane, 2,6,10-trimethyl-dodecane, linalool, and propanoic acid, octyl ester were identified after reheating. Hydrocarbons (2-methyltetracosane, dodecane) exhibited fatty characteristics while terpenes (3-carene, linalool, carveol) derived from spices contributed herbaceous and citrus-like flavors, enhancing the overall aroma and freshness of reheated meat [55]. VOCs generated at reheating produced a distinct flavor profile, contributed specific aromas to the meat, and retained the sensory profile of preheated stir-fried chicken. Among VOCs, 1-hepten-3-one, 3-furaldehyde, dimethyl phthalate, and maltol were only observed at the initial preheating temperature, and no further detection occurred.

The correlation between all volatile compounds detected in stir-fried chicken is illustrated in Figure 3D. It is interesting to note that VOCs were positively correlated with their own VOC group. For example, eucalyptol was positively correlated with maltol and terpiene-4-ol, which are alcohols, while it was negatively correlated with hydrocarbons (α-cubene, 3-carene). Similarly, propenal was positively correlated with nonanal and (E)-2-octenal and was negatively correlated with hydrocarbons. This could be because volatile compounds of the same chemical groups are similar in terms of thermal stability and have similar evaporation and retention rates [56].

**Figure 4:**
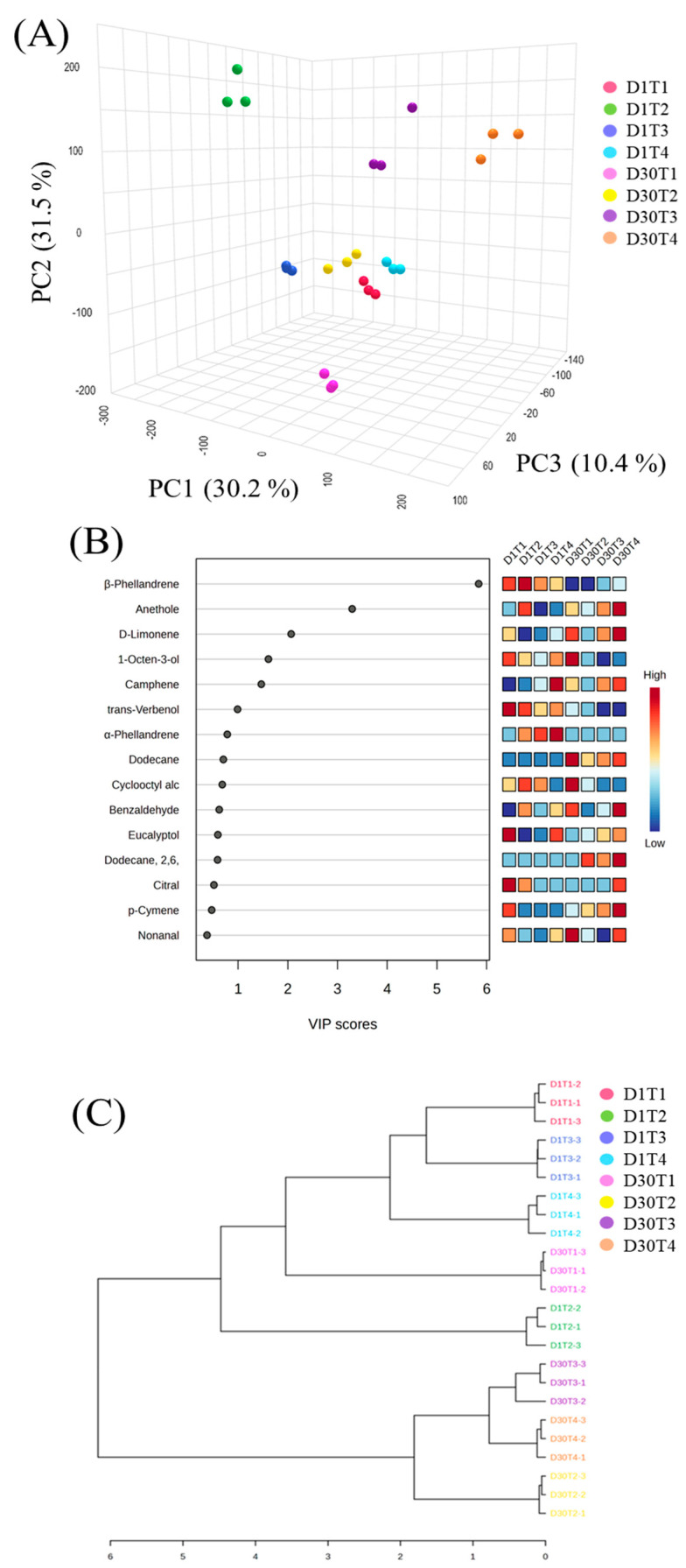
PLS-DA (**A**), VIP score (**B**), and dendrogram (**C**) of volatile compounds identified at four doneness levels (T1, T2, T3, T4) and heating methods (preheating (D1), reheating (D30). D1T1 = preheated medium rare; D1T2 = preheated medium; D1T3 = preheated medium well; D1T4 = preheated well done; D30T1 = reheated medium rare; D30T2 = reheated medium; D30T3 = reheated medium well; D30T4 = reheated well done.

The effect of preheating (D1) and reheating (D30) on VOC production was further evaluated by PLS-DA (A), based on VIP scores (B), and using a dendrogram (C) (Figure 4). PLS-DA showed that the first 3 PCs occupied 72.1% of the total variance, demonstrating a sufficient degree of explanation (Figure 4A). A scattered distribution of groups indicates a significant difference in the type and content of VOCs in doneness levels and heating process. The VIP score (Figure 4B) further evaluated the compounds with distinguished aromas at D1 and D30. A total of 6 VOCs scored more than 1, showing them to be potential markers for aroma formation in stir-fried chicken. Meanwhile, hydrocarbons and alcohols were found to be primary potential markers, contributing to 90% of all potential markers. The dendrogram represented a hierarchical relationship between heating and doneness levels, illustrating that doneness levels were closely associated with the amount of heating applied (Figure 4C). All reheating doneness levels except D30T1 formed a single hierarchy. In contrast, all preheating levels formed another hierarchy, showing a significant relationship between preheating and reheating in stir-fried chicken.

### 3.3. Texture Profile Analysis (TPA)

Table 1 shows the texture profile analysis (TPA) for preheating (D1) and reheating (D30) methods of stir-fried chicken. TPA depicted a significant difference (*p* < 0.05) at different doneness levels and during the heating process. Results showed that hardness decreased with the increase in doneness levels, whereas T3 (medium well) showed significantly reduced hardness among all the doneness levels. The decrease in hardness was due to the gelatinization of collagen and the relaxation of the muscle fibers after heating was applied [57]. Moreover, reheating showed considerably lower hardness values at all doneness levels. The decreased hardness (5921.80 g) seen at higher doneness level (D30T4) could be attributed to the redistribution of water inside meat during freeze storage and the gelatinization of collagen. Dick et al. [58] showed similar results, where the reheating of beef products showed decreased hardness values compared to preheating. However, the product takes longer to reheat (15 min at 100 °C) due to beef’s more tightly bound muscle fibers and its greater amounts of collagen than chicken. Wang et al. [34] reheated a Hongsu chicken dish (72 °C) using three reheating methods (water boiling, steaming, microwave, and oven roasting) and found reduced hardness, with the most significant reduction occurring for water boiling. No significant differences (*p* > 0.05) were observed for cohesiveness and springiness for any doneness level or heating process. Meanwhile, gumminess and chewiness also had no significant differences between preheating and reheating except for the medium well doneness level, which showed a significantly reduced value. This could be because cooking led to the rupture of the meat structure, the hydrolysis of collagen, and protein degradation, eventually softening the chicken cubes [59]. Additionally, water retention inside the meat after preheating could be responsible for the improved TPA of reheated meat [57]. The uniform texture and reduced hardness achieved in preheating could lead to a standardized quality of pre-cooked chicken in industries. Additionally, the nutritional content could be preserved while saving time and energy.

### 3.4. Instrumental Color Measurement

Color is a quality indicator for the consumption or rejection of meat. Doneness levels influenced the lightness (L*), redness (a*), and yellowness (b*) of stir-fried chicken (Table 2). No significant difference was observed in the L* doneness levels, except for D30T4 (48.14). A similar trend was followed by a*, where D1T4 (10.29) and D30T4 (9.52) showed reduced redness, whereas b* increased with the increase in doneness levels. The results indicated that preheating and reheating preserved the L* and a* of the meat, while alleviating b*. This could be because reheating precooked meat reduced the conversion of met-myochromogen from oxy-myoglobin and met-myoglobin [60]. The hindrance of water evaporation by plastic bags during reheating saw the moisture content retained; hence, the color remained unchanged. Moreover, the denaturation of haem pigment and other meat proteins increases b* in the meat [61]. Additionally, lipid oxidation contributed to the secondary oxidative products (e.g., MDA), and the reduction of oxy-myoglobin to met-myoglobin resulted in increasing b* values. These findings correlate with the TBARS results (Figure 2D). It is worth noting that the change in color at a well done doneness level (T4) could be attributed to the formation of browning pigments during the Maillard reaction, and the intensity of the Maillard reaction is proportional to the temperature applied for cooking [62].

### 3.5. Sensory Evaluation

**Figure 5:**
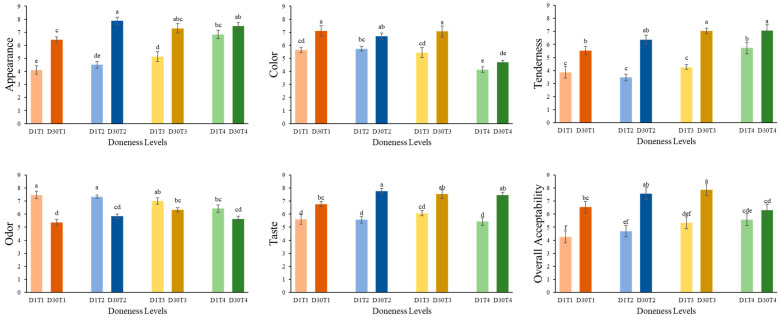
Sensory analysis of preheating (D1) and reheating (D30) at four doneness levels. Lowercase letters (a, b, c, d, e, f) showed a significant difference (*p* < 0.05) among four doneness levels. D1T1 = preheated medium rare; D1T2 = preheated medium; D1T3 = preheated medium well; D1T4 = preheated well done; D30T1 = reheated medium rare; D30T2 = reheated medium; D30T3 = reheated medium well; D30T4 = reheated well done.

Sensory evaluation was performed to assess the comprehensive quality attributes of the food [63]. Figure 5 shows the sensory analysis of meat at the preheating (D1) and reheating (D30) of stir-fried chicken at different doneness levels. The heating process significantly influenced the sensory analysis of stir-fried chicken, with improved sensory scores observed after reheating. Statistically significant (*p* < 0.05) results were obtained for both doneness levels and the heating process. Higher appearance scores were observed for T2 and T3 doneness levels for both preheating (D1) and reheating (D30). The color became homogeneous and shiny until T3 (medium well), and then turned uneven and black at T4, indicating that increased heating has a negative impact on color. This is consistent with the results shown in Table 2. However, better tenderness was seen after reheating, and the bad odor was reduced. Moreover, the present research results indicated that the increased doneness levels improved mastication and juiciness. The taste shifted from medium to strong in the change from preheating to reheating. Overall acceptability assessents revealed that D1T3 and D30T3 samples showed higher acceptability, with reheated samples (D30T3) exhibiting an appetizing smell and plentiful flavor that made the food more acceptable. Wang et al. [34] reheated Hongsu chicken using different cooking methods and observed increased sensory attributes after reheating during analysis. Similarly, Li et al. [38] reheated braised beef with potatoes and found better results. However, acceptability decreased at well done doneness due to reduced tenderness and an off odor. This might be because reheating well done cooked meat could lead to the further elution of moisture, making the chicken excessively tough and dry, as shown in TPA results (Table 1) [21].

### 3.6. Changes in Protein Digestibility of Stir-Fried Chicken

Protein digestibility is essential as it provides amino acids, which are the building blocks for various body functions, such as muscle repair, enzyme production, and hormone synthesis [17]. Protein digestibility was categorized in terms of gastric and intestinal digestibility with the addition of pepsin only and pepsin and trypsin together [2]. A statistically significant (*p* < 0.05) difference was observed for preheating (D1) and reheating (D30) methods (Figure 6). Additionally, for both preheating and reheating methods, increased (*p* < 0.05) gastric and intestinal protein digestibility was observed between doneness levels, except with well done doneness. For gastric digestibility, 30–55% digestibility was observed, with an increase up to 80% for intestinal digestibility. For T3, which is medium well heating, the digestibility ranged between 50 and 80%, showing improved digestibility. The increase in intestinal digestibility could be due to the cleavage of peptide bonds and the synergistic effect of pepsin and trypsin in proteolysis [64]. Overall, reheating resulted in higher digestibility than preheating, where D30T3 showed better gastric and intestinal digestibility. This might be due to enhanced protein carbonylation and oxidation processes, which are highly correlated with the digestibility of meat [65]. Additionally, the gelatinization of collagen protein and improved TPA (Table 1) resulted in increased digestibility after reheating. Similar results were obtained by Ferreira et al. [16], where reheated chicken patties showed increased digestibility compared to cooked and chilled chicken patties. Xu et al. [44] studied processed chicken breast meat containing pork fat replacers and found a decrease in digestibility after oven reheating, possibly due to increased surface hydrophobicity. Ramos et al. [66] demonstrated that the change in protein secondary structure could significantly affect the sensitivity of protein to digestive enzymes. Therefore, the lowered content of α-helix, as shown in Figure 2F, resulted in increased protein digestibility, which was further supported by the findings of Jiang et al. [67].

### 3.7. Microstructural Alterations in Stir-Fried Chicken

Figure 7 illustrates the microstructure of stir-fried chicken during preheating and reheating across four doneness levels. Heating significantly altered the meat’s microstructure, and muscle fibers exhibited noticeable boundaries at the initial preheating stage (D1T1). Myofibrillar proteins began to contract as heating progressed, while sarcoplasmic proteins aggregated (D1T2), leading to slightly compacted muscle fibers. At medium well doneness (D1T3), the substantial shrinkage of muscle fibers was observed, accompanied by the thermal denaturation of collagen between muscle bundles. This resulted in the formation of water channels and more pronounced shrinkage, which negatively affected the water-holding capacity of the muscle [68]. At the well done doneness level (D1T4), the arrangement of sarcoplasmic and myofibrillar proteins became disordered, causing significant moisture loss and structural damage, as reported by Li et al. [69]. Furthermore, novel structures and channels of varying sizes were observed at high temperatures, affecting meat tenderness due to the reorganization of the microstructure [37].

Upon reheating to medium rare (D30T1) doneness, the shrinkage of muscle fibers was evident and became more pronounced with continued heating. The extracellular muscle bundle boundaries blurred, and protein coagulation intensified at medium (D30T2) and medium well (D30T3) reheating doneness levels. Reheating preheated well done meat (D30T4) resulted in the additional shrinkage and hardening of muscle fibers, characterized by denatured dense muscle bundles and irregularly large spaces [70]. These results indicate that preheating and reheating progressively destroy the microstructure of myofibrillar proteins as doneness levels increase. This agrees with the findings of SH and SS content (as mentioned in Figure 2B,C).

### 3.8. Correlation Analysis

**Figure 8:**
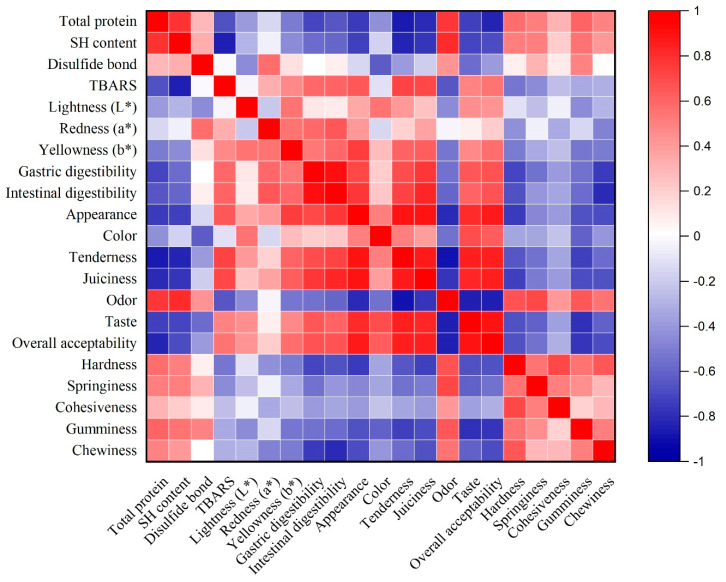
Correlation analysis of oxidation, quality, and digestibility parameters.

To further explore the relationship between oxidation, quality, and digestibility indicators, correlation analysis was conducted (Figure 8). The results revealed a strong positive correlation between gastric and intestinal digestibility and most sensory parameters, except for odor, which decreased as digestibility increased. Additionally, digestibility showed a negative correlation with the TPA values, indicating that reduced hardness enhances meat digestibility. Conversely, the protein profile parameters (total protein, SH content, and disulfide bonds) exhibited a positive correlation with TPA values, suggesting that protein alteration influences meat’s texture.

## 4. Conclusions

Preheating and reheating play a significant role in improving the quality and digestibility of stir-fried chicken. In this study, the raw chicken breast was diced and stir-fried (preheating, D1) at four doneness levels: medium rare, medium, medium well, and well done. Cooked samples were stored at −18 °C for 30 days and subsequently boiled (reheating, D30) to an internal temperature of 72 °C. A total of 59 VOCs were generated. Among these VOCs, β-phellandrene, camphene, 1-octen-3-ol, and α-phellandrene were key VOCs in in preheating, while 1-octene-3-ol, anethole, d-limonene, and benzaldehyde were key VOCs in reheating. The total protein (49.43–41.81 g/L) and SH content (93.74–77.65 µmol/mL) decreased during preheating and reheating with the increase in doneness levels, while disulfide bond detection was only lowered (29.19 µmol/mL) in reheating. Moreover, lipid oxidation increased with the increase in doneness levels. Texture analysis revealed reduced hardness, chewiness, and gumminess values, with a significant reduction seen during reheating. However, lightness (L*) and redness (a*) statistically remained the same, except at well done doneness level (T4), while yellowness (b*) increased with the increase in doneness levels. Moreover, sensory evaluation showed better results for D30T3 samples. Gastric digestibility ranged from 30 to 55%, while intestinal digestibility improved by up to 80% in reheating, with D30T3 depicting the highest digestibility. Microstructure analysis uncovered that the highest structural deformation occurred at the well done (T4) doneness level. Overall, preheating (D1), followed by reheating (D30), enhanced meat quality, flavor, and digestibility, with T3 (medium well) demonstrating better texture, color, sensory evaluation, and protein digestibility. This study examined the impact of preheating and reheating on stir-fried chicken. The application of these findings in the food sector is recommended to achieve well-textured, highly digestible flavored meat with minimal nutritional changes. Additionally, investigating the effect of storage methods on stir-fried chicken cooked at different doneness levels could provide valuable insights.

## Figures and Tables

**Table 1:** Effect of preheating and reheating on texture profile analysis (TPA).

	Samples	Hardness (g)	Gumminess	Cohesiveness	Chewiness	Springiness
Preheating	D1T1	7180.06 ± 373.08 ^a^	3148.32 ± 189.30 ^a^	0.60 ± 0.04 ^a^	2880.58 ± 184.01 ^a^	0.62 ± 0.03 ^a^
	D1T2	6531.05 ± 447.80 ^b^	3023.48 ± 247.87 ^ab^	0.57 ± 0.04 ^a^	2593.12 ± 133.32 ^ab^	0.61 ± 0.03 ^a^
	D1T3	6072.00 ± 421.12 ^cd^	2918.03 ± 202.88 ^ab^	0.56 ± 0.03 ^a^	2506.56 ± 152.52 ^ab^	0.60 ± 0.04 ^a^
	D1T4	6424.01 ± 478.96 ^b^	3167.35 ± 143.01 ^a^	0.57 ± 0.04 ^a^	2654.79 ± 134.13 ^ab^	0.60 ± 0.03 ^a^
Reheating	D30T1	6310.62 ± 427.44 ^bc^	2751.69 ± 247.17 ^ab^	0.57 ± 0.02 ^a^	2714.24 ± 182.95 ^ab^	0.57 ± 0.03 ^a^
	D30T2	5914.67 ± 482.57 ^d^	2442.86 ± 217.18 ^ab^	0.56 ± 0.02 ^a^	2411.37 ± 163.37 ^bc^	0.58 ± 0.02 ^a^
	D30T3	5365.85 ± 427.47 ^e^	2383.84 ± 243.82 ^c^	0.55 ± 0.03 ^a^	2204.27 ± 172.43 ^c^	0.56 ± 0.02 ^a^
	D30T4	5921.80 ± 504.45 ^d^	2800.01 ± 194.19 ^ab^	0.56 ± 0.02 ^a^	2599.75 ± 181.33 ^abc^	0.58 ± 0.02 ^a^

D1T1 = preheated medium rare; D1T2 = preheated medium; D1T3 = preheated medium well; D1T4 = preheated well done; D30T1 = reheated medium rare; D30T2 = reheated medium; D30T3 = reheated medium well; D30T4 = reheated well done. Superscripts (a, b, c, d, e) within a column indicate the presence of significant differences (*p* < 0.05) among the four doneness levels.

**Table 2:** Effect of preheating and reheating on color (L*, a*, b*) values.

	Sample	L*	a*	b*
Preheating	D1T1	56.75 ± 1.82 ^ab^	14.54 ± 1.31 ^a^	25.16 ± 0.90 ^e^
	D1T2	58.41 ± 2.48 ^a^	13.46 ± 0.50 ^ab^	27.27 ± 1.26 ^de^
	D1T3	59.10 ± 1.93 ^a^	12.99 ± 0.88 ^ab^	29.72 ± 1.62 ^cd^
	D1T4	53.25 ± 1.75 ^b^	10.29 ± 1.03 ^c^	31.95 ± 2.15 ^ab^
Reheating	D30T1	55.11 ± 1.89 ^ab^	13.64 ± 0.52 ^ab^	27.90 ± 0.90 ^cde^
	D30T2	57.09 ± 2.40 ^ab^	12.50 ± 0.44 ^ab^	28.42 ± 1.41 ^cde^
	D30T3	57.43 ± 1.13 ^ab^	12.32 ± 1.18 ^b^	30.99 ± 1.24 ^abc^
	D30T4	48.14 ± 1.93 ^c^	9.52 ± 1.11 ^c^	33.38 ± 1.85 ^a^

D1T1 = preheated medium rare; D1T2 = preheated medium; D1T3 = reheated medium well; D1T4 = preheated well done; D30T1 = reheated medium rare; D30T2 = reheated medium; D30T3 = reheated medium well; D30T4 = reheated well done. Superscripts (a, b, c, d, e) within a column indicate the presence of significant differences (*p* < 0.05) among four doneness levels.

## Data Availability

The data presented in this study are available on request from the corresponding author. The data are not publicly available due to privacy restrictions.

## Supplementary Materials

The following supporting information can be downloaded at. https://www.mdpi.com/article/10.3390/foods14040643/s1. Figure S1: Radar map (A) and Venn diagram (B) of volatile compounds identified at preheating (D1) and reheating (D30). D1T1 = preheated medium rare; D1T2 = preheated medium; D1T3 = preheated medium well; D1T4 = preheated well done; D30T1 = reheated medium rare; D30T2 = reheated medium; D30T3 = reheated medium well; D30T4 = reheated well done. Table S1: Sensory evaluation Performa for stir-fried chicken.
