# Supplementary material for: Optimizing Protein Profile, Flavor, Digestibility, and Microstructure: The Impact of Preheating and Reheating in Stir-Fried Chicken
Source: Foods. 2025 Feb 14;14(4):643. doi: 10.3390/foods14040643 (PMC11854888; doi:10.3390/foods14040643)
Supplement: Supplementary file 1 [file foods-14-00643-s001.zip › foods-3427701-supplementary.pdf]

Supplementary Table S1. Sensory evaluation Performa for stir-fried chicken.

Evaluation Indicators	Description	Score
Appearance	Loose, rough, poor compactness	0<x≤2
	Uneven, slight loose, high Juice exudation	2<x≤4
	Slightly less firmness, slight juice exudation	4<x≤6
	Flat, slight juice exudation	6<x≤8
	Flat, firm, no juice exudation	8<x≤10
Color	Dark black, uneven, dull	0<x≤2
	Brown, uneven, dull	2<x≤4
	Brown, homogeneous and shiny	4<x≤6
	Tan, even, shiny	6<x≤8
	Reddish-brown, homogeneous and shiny	8<x≤10
Tenderness	The meat is coarse and old	0<x≤2
	The meat quality is average	2<x≤4
	The meat is tender	4<x≤6
	The meat is relatively tender	6<x≤8
	The meat is highly tender	8<x≤10
Odor	Mild, odorous	0<x≤2
	Mild, no bad odor	2<x≤4
	Average, no bad odor	4<x≤6
	More intense odor	6<x≤8
	Intense and long-lasting odor	8<x≤10
Taste	Weak	0<x≤2
	Slight identifiable	2<x≤4
	Medium	4<x≤6
	Strong	6<x≤8
	Very strong	8<x≤10
Overall acceptability	Not accepted	0<x≤2
	Reluctantly accepted	2<x≤4
	Comparatively accepted	4<x≤6
	Easily accepted	6<x≤8
	Highly accepted	8<x≤10

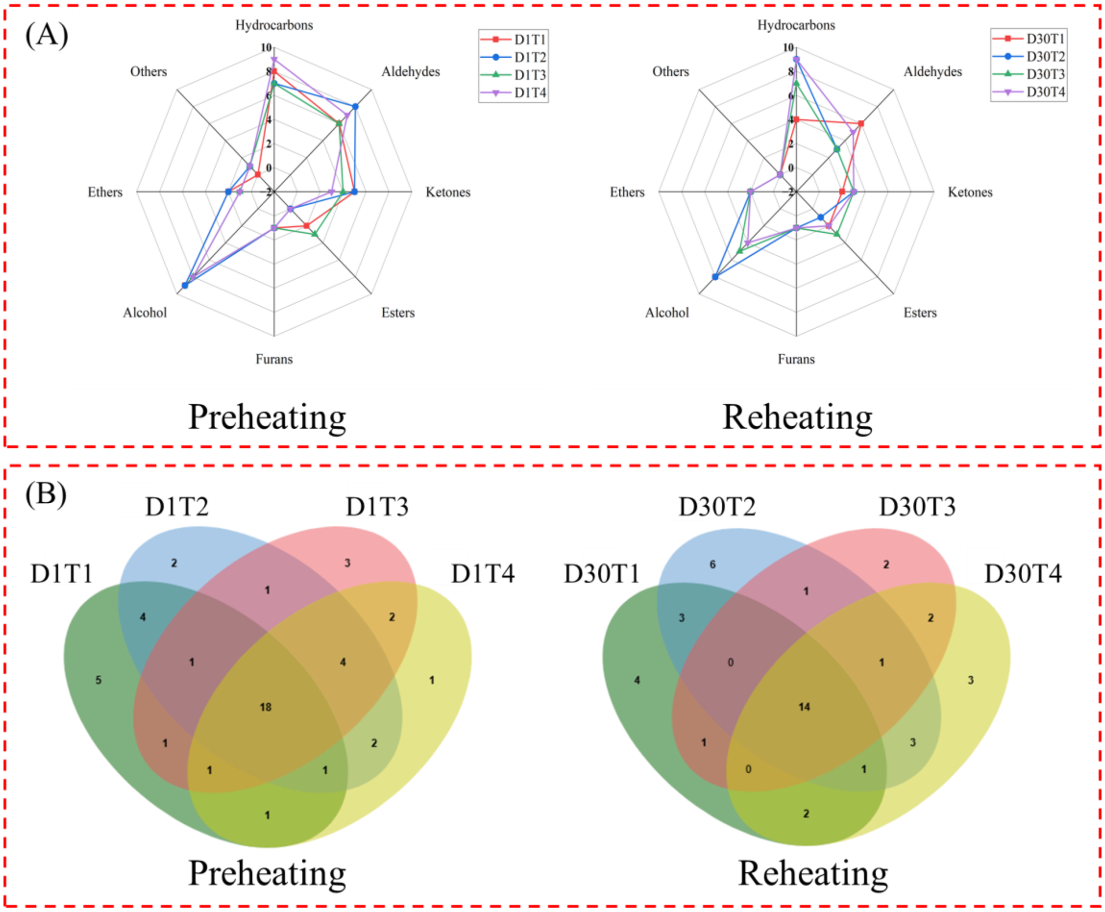

Supplementary Figure S1. Radar map (A) and Venn diagram (B) of volatile compounds identified at preheating (D1) and reheating (D30). D1T1= Preheated medium rare; D1T2= Preheated medium; D1T3= Preheated medium well; D1T4= Preheated well done; D30T1= Reheated medium rare; D30T2= Reheated medium; D30T3= Reheated medium well and D30T4= Reheated well done.
